# Ribonuclease-1 treatment after traumatic brain injury preserves blood–brain barrier integrity and delays secondary brain damage in mice

**DOI:** 10.1038/s41598-022-09326-2

**Published:** 2022-04-06

**Authors:** Tobias J. Krämer, Per Hübener, Bruno Pöttker, Christina Gölz, Axel Neulen, Tobias Pantel, Hermann Goetz, Katharina Ritter, Michael K. E. Schäfer, Serge C. Thal

**Affiliations:** 1 Department of Anesthesiology, University Medical Center of Johannes Gutenberg University, Langenbeckstrasse 1, 55131 Mainz, Germany; 2 Department of Neurosurgery, University Medical Center of Johannes Gutenberg University, Langenbeckstrasse 1, 55131 Mainz, Germany; 3 Cell Biology Unit, University Medical Center of Johannes Gutenberg University, Langenbeckstrasse 1, 55131 Mainz, Germany; 4 Focus Program Translational Neurosciences, University Medical Center of Johannes Gutenberg University, Langenbeckstrasse 1, 55131 Mainz, Germany; 5 Research Center for Immunotherapy, University Medical Center of Johannes Gutenberg University, Langenbeckstrasse 1, 55131 Mainz, Germany; 6 Center for Molecular Surgical Research, University Medical Center of Johannes Gutenberg University, Langenbeckstrasse 1, 55131 Mainz, Germany; 7 Department of Anesthesiology, Helios University Hospital Wuppertal, University Witten/Herdecke, Heusnerstrasse 40, 42283 Wuppertal, Germany; 8 Faculty of Health, University Witten/Herdecke, Witten, Germany

**Keywords:** Brain injuries, Neuroscience, Blood-brain barrier, Cell death in the nervous system

## Abstract

Traumatic brain injury (TBI) involves primary mechanical damage and delayed secondary damage caused by vascular dysfunction and neuroinflammation. Intracellular components released into the parenchyma and systemic circulation, termed danger-associated molecular patterns (DAMPs), are major drivers of vascular dysfunction and neuroinflammation. These DAMPs include cell-free RNAs (cfRNAs), which damage the blood–brain barrier (BBB), thereby promoting edema, procoagulatory processes, and infiltration of inflammatory cells. We tested the hypothesis that intraperitoneal injection of Ribonuclease-1 (RNase1, two doses of 20, 60, or 180 µg/kg) at 30 min and 12 h after controlled-cortical-impact (CCI) can reduce secondary lesion expansion compared to vehicle treatment 24 h and 120 h post-CCI. The lowest total dose (40 µg/kg) was most effective at reducing lesion volume (− 31% RNase 40 µg/kg vs. vehicle), brain water accumulation (− 5.5%), and loss of BBB integrity (− 21.6%) at 24 h post-CCI. RNase1 also reduced perilesional leukocyte recruitment (− 53.3%) and microglial activation (− 18.3%) at 120 h post-CCI, but there was no difference in lesion volume at this time and no functional benefit. Treatment with RNase1 in the early phase following TBI stabilizes the BBB and impedes leukocyte immigration, thereby suppressing neuroinflammation. RNase1-treatment may be a novel approach to delay brain injury to extend the window for treatment opportunities after TBI.

## Introduction

Mechanical stress to brain tissue induces necrosis and apoptosis (termed primary injury), resulting in the release of cellular contents into the brain parenchyma and blood circulation^1^. Some intracellular biomolecules, including cell-free/extracellular ribonucleic acids (cfRNAs/eRNAs) act as damage-associated molecular patterns (DAMP) with potent pro-inflammatory activity^2^. cfRNA is present in plasma as nucleotide chains that are not bound to other molecules, is bound to proteins, or is stored in extracellular membrane vesicles^3,4^. In the latter two cases, cfRNA is protected from degradation by RNases^5^. These cfRNAs are abundant in the extracellular milieu after traumatic brain injury (TBI)^2^, where they promote vascular endothelial grow factor (VEGF) activation and ensuing disruption of the blood blood–brain barrier (BBB), leading to edema and immune cell infiltration^6^. These delayed processes are central drivers of the more expansive secondary damage following local TBI^7–9^. Cell-free RNA has been reported to promote intercellular adhesion molecule-1 (ICAM-1)-dependent endothelial adhesion and extravasation of leukocytes^10^. Further, sequence-specific cfRNA binding to toll-like receptors (TLRs, such as TLR7, 8, and 12) activates NF-κB pathway signaling and upregulates production of the pro-inflammatory cytokine tumor necrosis factor-α (TNF-α) by macrophages and microglia^11,12^. Extracellular RNAs have also been shown to increase expression of cytokines by macrophages via TLR3^13,14^, enhance interferon-γ expression by astrocytes and microglia^15,16^, support the maturation of dendritic cells^17^, and exacerbate apoptosis via TLR3^18,19^. Several animal studies that have examined the efficacy of immunomodulatory agents in improving outcomes after TBI have yielded positive results^20,21^. Thus, elimination of cfRNAs at the site of injury or in the circulation could prevent secondary injury after TBI by stabilizing the BBB and promoting immunosuppression^22,23^.

The half-life of circulating cfRNA is dependent on plasma ribonuclease levels^24^. Ribonuclease-1 (RNase1) is a member of the RNase-A superfamily expressed in exocrine pancreas^25^, brain^26^, and in vascular endothelial cells^27,28^. The present study was designed to examine if RNase1 treatment post-TBI reduces brain edema, preserves BBB integrity, reduces brain lesion size, quells the neuroinflammatory response, and (or) improves functional outcome at 24 and 120 h after controlled-cortical-impact (CCI) in mice.

## Results

**Figure 1:**
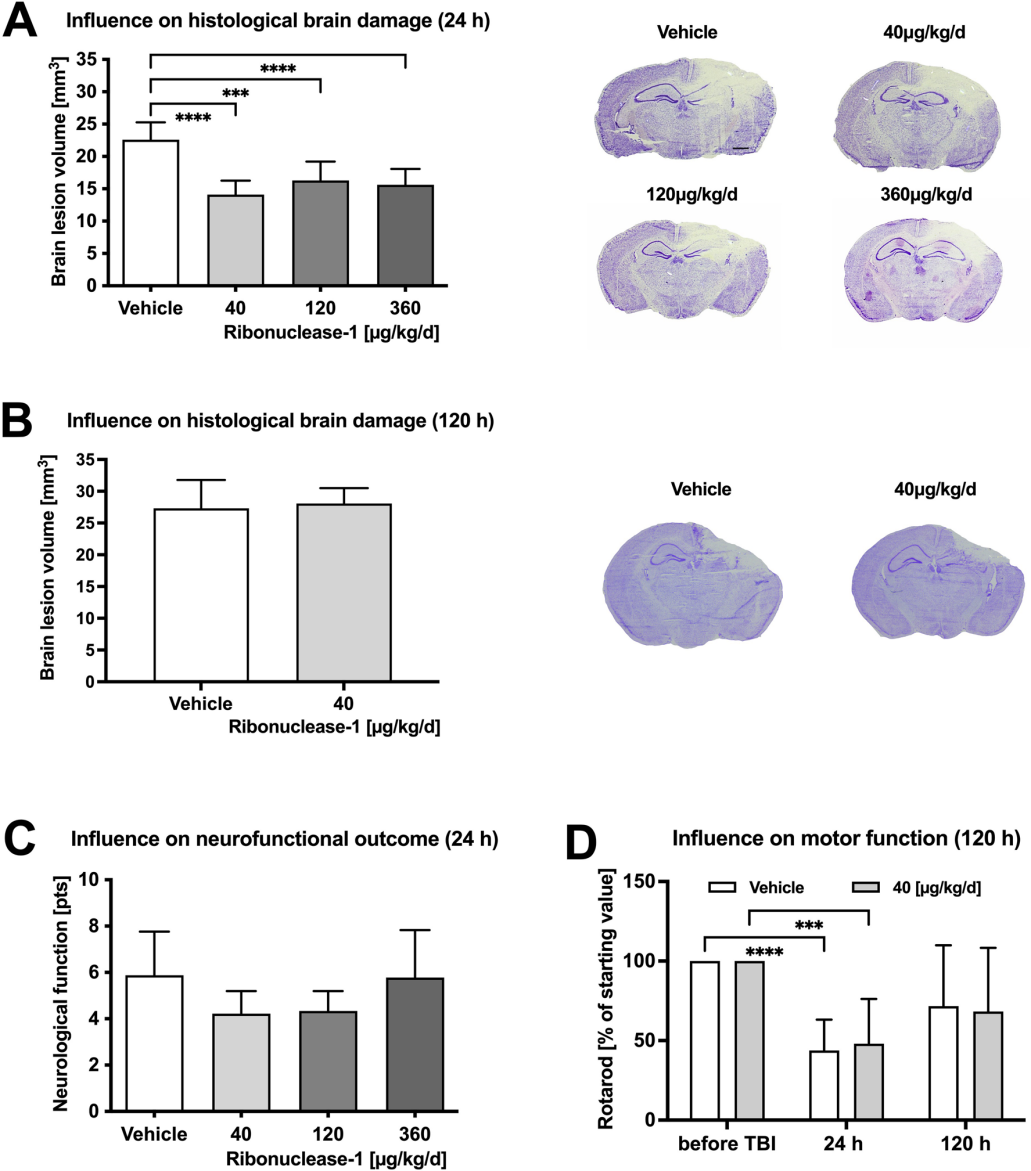
Influence on brain lesion and functional outcome. Brain lesion was determined 24 h (**A**) after controlled cortical impact injury in animals treated with RNase1 40, 120, 360 μg/kg/d or vehicle-solution (NaCl 0.9%, n = 10 each) by intraperitoneal injection 30 min and 12 h after brain insult. Treatment significantly reduced brain lesion volume in all treatment groups. With equal regime and amount of RNase1 120 h after CCI (**B**) impact lesion volume revealed no persistent treatment success. Neurofunctional deficits were determined at 24 h (**C**) and showed no effects on outcome with low dose showing the best score but failing to reach level of significance. The motoric function (**D**) was severely affected after 24 h, and animals recovered mainly after 120 h without significant difference between treatment and vehicle (n = 10/group). Data are presented as mean ± standard deviation; P values are adjusted for multiple comparisons by Sidak correction. The figure was generated with GraphPad Prism 9.0.

Animals were randomly assigned to receive a low-dose of RNase1 (LD: 2 × 20 μg/kg, n = 10), an intermediate-dose of RNase1 (MD: 2 × 60 μg/kg, n = 10), a high-dose of RNase1 (HD: 2 × 180 μg/kg, n = 10), or vehicle (2 × 500 μL NaCl 0.9%, n = 10) by i.p. injection 30 min and 12 h post-CCI. Contusion volume increase by 60% within 24 h, indicating induction of secondary injury^29,30^. However, all RNase1 doses significantly reduced lesion volume compared to vehicle treatment (vehicle: 22.6 ± 2.7 mm^3^; LD: 14.1 ± 2.2 mm^3^; MD: 16.3 ± 2.9 mm^3^; HD: 15.6 ± 2.4 mm^3^; all *p* < 0.001; Fig. 1A). Lesion volumes continued to increase in all groups up to 120 h post-CCI and stabilized thereafter. Thus, post-CCI treatment with RNase1 can delay but not completely prevent brain damage (vehicle: 27.3 ± 4.4 mm^3^; LD: 28.1 ± 2.4 mm^3^; Fig. 1B). Neurofunctional impairment was assessed by a deficit score ranging from 0 (no motor deficits) to 15 (severe impairment) according to the criteria of Timaru-Kast and colleagues^34^. There were no significant differences in scores among groups at 24 h post-CCI, although low-dose RNase1 treatment resulted in an overall reduction compared to vehicle (4.5 ± 1.3 vs. 5.9 ± 1.9, *p* = 0.0658) (Fig. 1C). Prior to trauma, the animals averaged 61.3 s in the acceleration task on the rotarod and up to an average speed of 5.7 rounds per minute. There were no differences in motor function as assessed in the rotarod task at 120 h post-CCI. (Fig. 1D).

**Figure 2:**
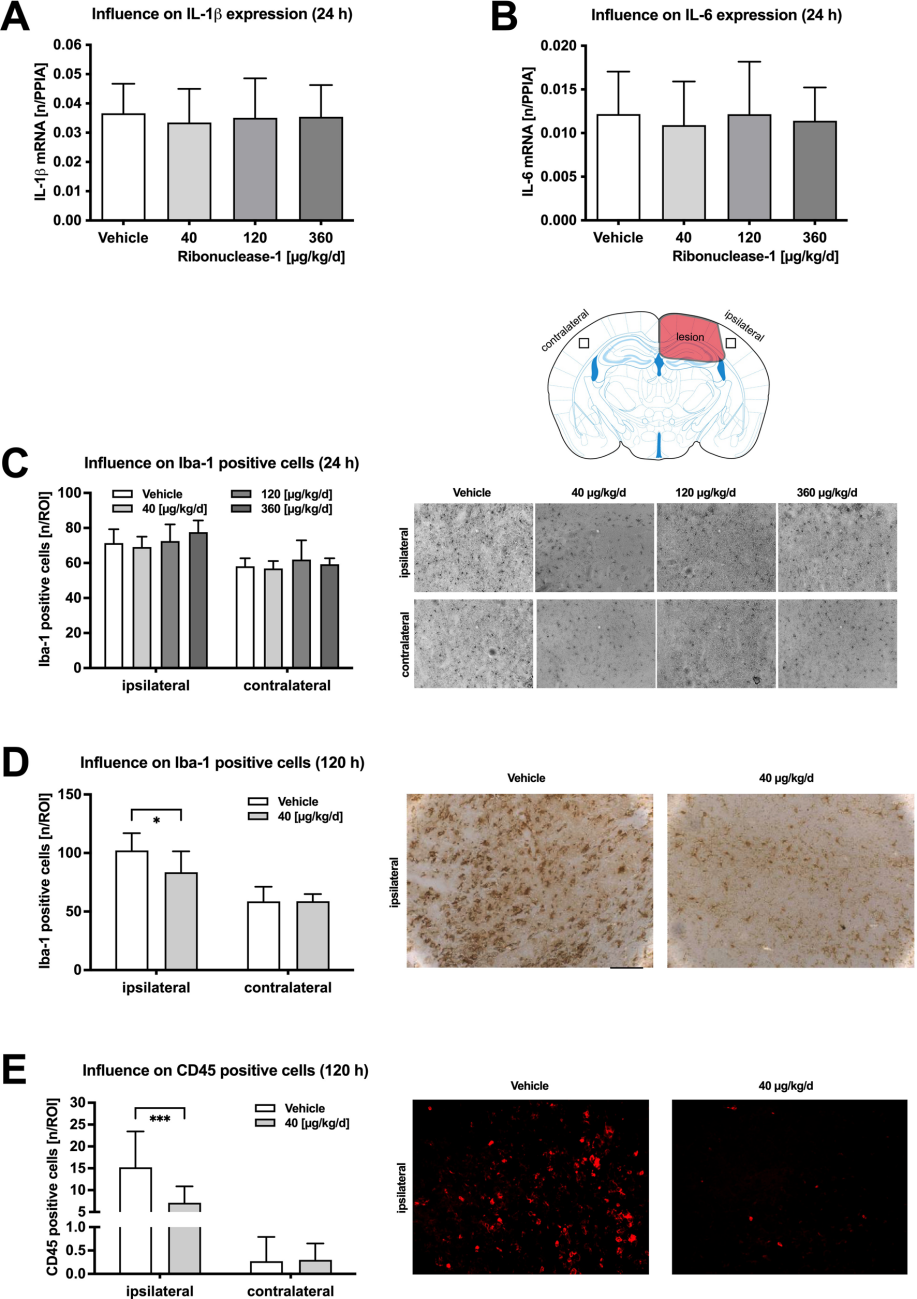
Regulation of markers for inflammation. Expression of inflammatory marker genes IL-1 $\beta$ (**A**) and IL-6 (**B**) were quantified and showed no treatment dependent effect. As marker for microglia activation the number of Iba-1 positive cells (**C**) were quantified and showed no difference between treatment (n = 10 each) groups 24 h after CCI. Iba-1 positive cells were quantified at 24 h and 120 h, and CD45-positive cells at 120 h after trauma using the same standardized region of interest as shown in the overview image (**C**). Contralateral number of Iba-1 positive cells remained identical 120 h after trauma (**C**). Pericontusional increase of Iba1- cells was significantly lower in the RNase1 treated mice (**D**) linked to a significant decrease of CD45 positive cells (**E**) in the RNase group (n = 10/group) 120 h after CCI. Data are presented as mean ± standard deviation; P values are adjusted for multiple comparisons by Sidak correction. The figure was generated with GraphPad Prism 9.0.

To examine if RNase1 treatment suppresses neuroinflammation, mRNA expression levels of interleukin (IL)-1β (Fig. 2A) and IL-6 (Fig. 2B) were determined in brain samples from the lesioned (ipsilateral) hemisphere by qPCR at 24 h post-CCI. No differences were present between RNase1 or vehicle treated animals. In addition, the number of activated microglia in the ipsilateral hemisphere as indicated by Iba-1 immunostaining was elevated in all treatment groups compared to the corresponding contralateral hemisphere and did not differ among groups at 24 h post-CCI (Fig. 2C). In contrast, cerebral inflammation was enhanced in the ipsilateral hemisphere at 120 h post-CCI, and both the numbers of Iba-1 + and CD45 + cells were significantly reduced by RNase1 treatment (Iba-1 + , *p* = 0.029; CD45 + , *p* = 0.01) (Fig. 2D,E).

**Figure 3:**
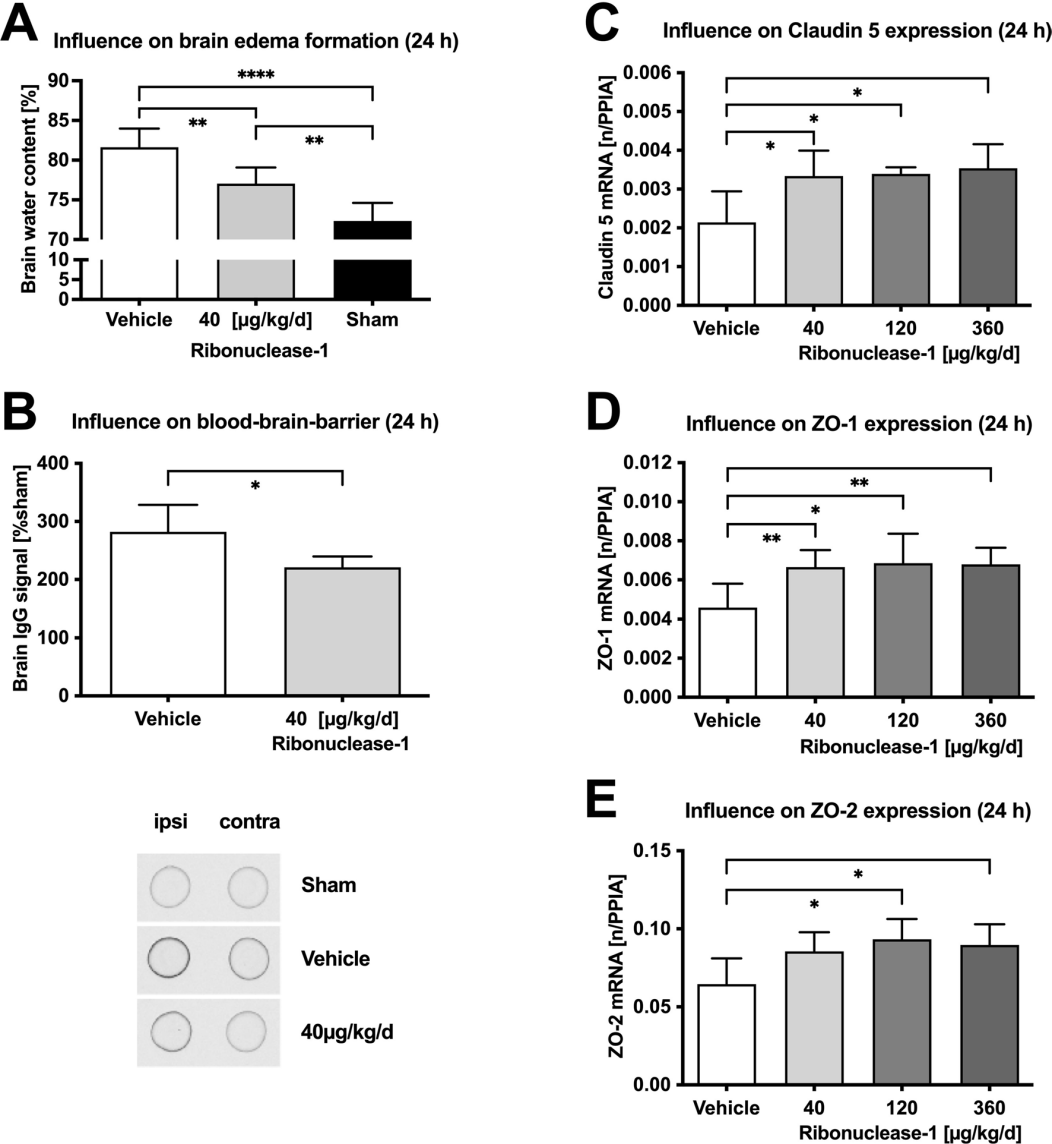
Influence on cerebral edema und regulation of tight junction protein mRNAs. Brain water content determined 24 h after CCI (**A**) was significantly lower with 40 μg/kg/d RNase1 compared to vehicle solution (n = 10 each). Integrity of the blood-brain barrier was investigated 24 h after lesion by quantification of immunoglobulin G (IgG) extravasation (**B**), which was significantly lower in RNase treated animals (n = 10 each). 24 h after insult mRNA expression of claudin 5 (**C**), ZO-1 (**D**), and ZO-2 (**E**) was quantified to investigate the impact of RNase treatment on VEGF regulated tight junction protein and showed significantly higher values with RNase treatment (n = 10 each). Data are presented as mean ± standard deviation; P values are adjusted for multiple comparisons by Sidak correction. The figure was generated with GraphPad Prism 9.0.

In a separate set of animals, brain water content and BBB permeability were compared between brain injured animals receiving vehicle or LD RNase1 treatment and sham operated animals with LD RNase1 treatment (n = 10 mice per group). LD was selected as the most effective dose from the previous experiments. First, the influence of LD RNase1 on post-CCI brain edema was examined by measuring the brain water content by vacuum drying. Treatment with RNase significantly reduced post-CCI brain water content from 81.6% ± 2.3% (contralateral: 74.9% ± 1.1%) to 77.1% ± 2.0%, p = 0.0027 (contralateral: 72.1% ± 2.3%), suggesting prevention of post-CCI edema. However, post-CCI water content was still significantly higher than contralateral or in the sham group (ipsilateral: 72.3% ± 2.3%; *p* < 0.001; contralateral: 73.6% ± 2.0; Fig. 3A), indicating only partial efficacy. To determine if LD RNase1 treatment also stabilizes BBB integrity, IgG extravasation was quantified as a surrogate parameter of functional integrity of the blood–brain barrier (BBB). These measurements revealed a 2–threefold higher signal in post-CCI tissue compared to sham operated animals (Fig. 3B) and a significant reduction by RNase1 treatment (40% lower IgG extravasation, *p* = 0.0073) compared to vehicle. To maintain BBB integrity, adequate structural support from tight-junction (TJ) proteins is essential. We therefore quantified the expression levels of mRNAs encoding the TJ proteins claudin-5, ZO-1, and ZO-2 by qPCR at 24 h post-CCI. mRNA expression levels of claudin-5 (+ 40%) and ZO-1 (+ 60%) were significantly higher in the LD RNase1 group (+ 60%) compared to vehicle-treated animals. ZO-2 mRNA level was significantly higher in the MD- (+ 43%) and HD-RNase1 (+ 38%) groups compared to vehicle-treated animals, whereas ZO-2 mRNA expression was not increased by LD-RNas1 treatment (Fig. 3C–E).

## Discussion

This is the first study demonstrating that RNase1 injection can preserve the blood–brain barrier integrity in the early period following TBI. Low-dose RNase1 upregulated multiple TJ protein mRNA levels, and reduced edema, inflammatory cell infiltration, macrophage activation and lesion volume. However, the treatment protocol (2 doses, at 30 min and 12 h post-contusion) was not sufficient for long-term reduction in lesion size, suggesting that pretreatment or prolonged post-treatment may be necessary. For instance, a protective effect against postoperative dementia and brain edema after stroke could only be achieved with RNase pretreatment^22,31^.

### Effects on brain edema

Vasogenic edema and IgG extravasation were reduced at one day post-TBI. The pathogenic effect of cfRNA may be partially attributed to reduced ZO-1 and claudin-5 expression^33^, while RNase1 can increase expression of ZO-1, ZO-2, and claudin-5, suggesting a stabilizing effect on BBB integrity. Under cerebral ischemia, vascular permeability is increased by cfRNA via the VEGF axis^6^. TBI leads to an upregulation^34^ or downregulation^35^ of VEGF and would be a possible candidate for more detailed analyses in future studies.

### Anti-inflammatory effects

In addition to VEGF signaling, cfRNAs stimulate the immune system via TLRs^11,12,18,36^, which induces the release of pro-inflammatory cytokines^10^ and shifts microglial phenotype to the pro-inflammatory M1 type^37,38^ known to induce neuronal damage and CNS dysfunction^39^. However, these anti-inflammatory actions appear to play only a minor role in the first 24 h post-CCI as there no alterations in IL-6 and IL-1β mRNA expression or microglial number, while significantly reduced numbers of Iba1 + and CD45 + cells were found at 120 h post-CCI. Due to the early beneficial effects on the BBB and immune system^10–19^, reduced neuroinflammation was still detected 120 h post-TBI. Collectively, these findings suggest that cfRNAs released during TBI induce leukocyte extravasation via VEGF2 activation^10^ and a simultaneous shift to the pro-inflammatory M1-phenotype^15^.

At 5 days after injury, brain lesion volume was similar between groups, suggesting that the initial putative beneficial effect was not long-lasting and not sufficient to results in functional improvement.

### Limitations of the study

These findings suggest that RNase1 treatment may be a useful adjunct therapy to reduce or delay secondary damage in the early phase post-TBI. Indeed, high cfRNA in systemic circulation is associated with poor outcome after TBI^23,40^. Further, RNase1 released from endothelial cells during ischemia^28,41^ may serve an endogenous protective function^42^. Thus, RNase1 treatment supplements an endogenous mechanism. RNase1 degrades ssRNAs but not extracellular rRNAs, mRNAs, or miRNAs. In a mouse model of myocardial infarction, RNAse1 treatment reduced myocardial edema and infarct size^42,43^. While this presumably reflects degradation of cfRNA, it is currently challenging to directly measure cfRNAs in small volumes of mouse plasma^31,43,44^. However, these protective effects of RNase1 treatment after TBI are consistent with suppression of pathways known to be activated by cfRNAs. RNase1 attenuated septic cardiomyopathy and cardiac apoptosis in a murine model of polymicrobial sepsis. Whereas there was no clear evidence of reduction in cfRNA nor a correlation between RNase1 and cfRNA levels^44^. After myocardial infarction in mice systemic application of RNase1 reduced edema formation and infarct size and improved survival. Plasma cfRNA levels were significantly increased compared with controls at 30 min after ligation, but no difference were present between treatment groups^43^. Exosomes^45^, microRNA^46^, or long non-coding RNA^47^ could possibly serve as indirect markers for cfRNA. Especially in in vivo studies with small rodents to overcome the limitation of limited plasma sample size.

Despite clear data on brain edema formation and early histological brain damage, the present study fails to provide evidence for functional improvement by RNase1 therapy after experimental TBI. Although the RNase1-treated animals have lower mean scores in the neuro-deficit score 24 h after CCI, these changes were not significant. Rotarod data failed to show any effect at 24 h and 5 days after injury. Reduction in extent of brain edema, blood–brain-barrier disruption and brain lesion at 24 h post injury therefore does not result in improved functional recovery. Furthermore, brain water levels were lower than expected and compared to recent other studies. Although the vacuum method is superior to the conventional oven technique in terms of quantifying water content in small samples^48^, the low water levels in all samples suggest a systemic measurement bias, which was equally evident in all experimental groups. Therefore, we decided to present the ipsi- and contralateral data to demonstrate the relative changes induced by RNase1 treatment.

In addition, only male animals are used in the study. We therefore cannot estimate the gender effect and the impact of RNase1 treatment in brain injured female mice. These effects need to be addressed in future studies.

To reduce the number of animals the study focused on the effect of RNase1 in brain injured animals. Therefore, the influence of RNAse1 in sham or healthy animals was not investigated in detail. Only in the study groups to determine brain-water content RNase1 was also given to sham animals. In these animals, neurological function was not negatively influenced by RNAse1 and were all 0 = normal (Figure S1). This indicates that RNase1 treatment does not have a highly negative impact on neurological function. Future studies should address in more detail the effect in sham or naïve animals to rule out any negative influence of RNase1.

## Conclusion

We demonstrate that i.p. RNase1 can stabilize the BBB following TBI, resulting in reduced vasogenic edema, leukocyte recruitment, and microglial activation in perilesional tissue. These data support a role for cfRNA and RNase1 in pathophysiology of TBI.

## Materials and methods

### Animals

This study was approved by the German animal protection committee (Landesuntersuchungsamt Rheinland-Pfalz, protocol number G-13-1-074). Experiments were performed in accordance with all national animal welfare guidelines and ARRIVE guidelines for reporting animal research^49^. The study was conducted using 90 male C57Bl6/N mice (Charles River Laboratories, Sulzfeld, Germany; weight: 22–28 g). Due to signs of severe stress, one animal in each of the two treatment arms (120 h survival time) was euthanized prior to the end of the observation period to prevent unnecessary suffering. The study protocol did not allow replacement of excluded animals.

### Drug preparation

Ribonuclease-1 (RNase1, Sigma, Germany) was dissolved in sterile normal saline by a third person not involved in the experiments or analysis, numbered and prepared for intraperitoneal (i.p.) injection. The experiments and the processing of the material was performed in blinded fashion. The syringes were covered to prevent identification of the solution and to ensure blinding of the person performing the injection. Mice were randomly allocated to receive two i.p. injections of 20, 60, or 180 µg/kg RNase1 or vehicle at 30 min and 12 h post-TBI. These doses are at least tenfold lower than required to induce adverse side effects in rodents^6,22,31^. To minimize the total number of animals used in accordance with ARRIVE guidelines^49^, no additional sham group was established for most experiments. Also, after the initial dose–response study, subsequent investigations of brain water accumulation, tissue IgG changes, and 120-h survival used only the most effective (low) dose of RNase1, with sham animals examined for comparison only when necessary. Sham animals received either LD-RNase1 solution.

### Traumatic brain injury

Traumatic brain injury was induced by CCI using an electromagnetic impact device (Leica Impact One™ Stereotaxic Impactor, Richmond, IL; tip: 3 mm; velocity: 6 m/s; duration: 200 ms; displacement: 1.5 mm). The settings were chosen to induce severe brain injury. Animals were anesthetized by intraperitoneal injection of 5 mg/kg midazolam, 0.05 mg/kg fentanyl, and 0.5 mg/kg medetomidine, an air/oxygen mixture (40% O_2_) was supplied via facemask in spontaneously breathing animals and body temperature was maintained at 37 °C rectal temperature with a feedback-controlled heating pad (Hugo Sachs, March-Hugstetten, Germany). Animals were fixed in a stereotactic frame (Kopf Instruments, Tujunga, USA) and a craniotomy (4 × 4 mm) was performed using a saline-cooled high-speed drill above the right parietal cortex between the sagittal, lambdoid, and coronal sutures and the insertion of the temporal muscle. Trauma was induced perpendicular and directly to right cortex by an experimenter blinded to treatment group allocation^50–52^. Thereafter, the craniotomy was closed with the initially removed bone, fixed with tissue glue (Histoacryl, Braun-Melsungen, Melsungen, Germany), the wound was closed with filament sutures and anesthesia was antagonized using 0.5 mg/kg Flumazenil and 2.5 mg/kg Atepamezol hydrochloride. Animals were allowed to recover for 1.5 h in a neonatal incubator (IC8000, Draeger, Luebeck, Germany) with controlled air temperature (35 °C) and ambient humidity (35%).

### Neurological outcome and Rotarod test

Before CCI (1 h) and at 24 h after CCI, an investigator blinded to experimental group assigned a neurologic severity score ranging from 0 (healthy) to 15 (severely impaired) (24 h groups: N = 40)^53^. An accelerating rotarod test (Rotarod Treadmill, MED Associates, INC, St Albans, VT) was conducted in the long-time surviving groups one day before and at 24 and 120 h post-CCI (N = 20). The rotarod speed was increased linearly from 4 to 40 rpm over 5 min. The investigation was completed when the mice fell off the rods. Briefly, each mouse was placed on an accelerating rotating cylinder, and the time and maximum speed at which the animal fell off (27 cm fall height) were recorded^54,55^. Four rotarod tasks were conducted before CCI and the mean value of each animal was defined as 100% “starting value”. 24 h and 120 h following injury, animals were tested in two trials per time point, averaged and evaluated relative to pre-injury latencies to correct for individual pre-injury performance.

### Tissue preparation, Nissl, Iba-1 and CD45 staining

Animals were deeply anesthetized by intraperitoneal injection of 5 mg/kg midazolam, 0.05 mg/kg fentanyl, and 0.5 mg/kg medetomidine. After cervical dislocation and decapitated, the brains were carefully excised, frozen in powdered dry ice, and stored at −20 °C. Coronal Sects. (10 μm) were cut at 500 μm intervals (24 h: n = 40 mice, 120 h: n = 20 mice), collected on Superfrost Plus Slides (Thermo Fisher, Waltham, MA, USA) and subjected to cresyl violet staining. The first section was taken at bregma + 3.14 mm according to the Mouse Brain Library atlas (http://www.mbl.org). Brain lesion volume was measured using a computerized image system (DeltaPix InSight, Smorum, Denmark)^56^. In brief, to control for the effect of brain edema, the area of uninjured brain tissue and the total area of the contralateral hemisphere were quantified in each section. Afterwards the injured area was calculated by subtraction of “normal” area in the injured hemisphere from total contralateral. Other slices were immunostained for the activated microglial marker Iba-1 using an anti-rabbit Iba-1 antibody (1:1500, Wako Pure Chemical Industries, Osaka, Japan) and a biotinylated anti-rabbit IgG (Vector Laboratories Inc., Burlingame, CA). Signals were detected using ABC Complex (Vector) and DAB (Thermo-Fischer, Waltham, MA). The appearance of Iba-1 + /CD45 + cells 120 h after lesion induction was quantified by dual staining using rat anti-CD45 (1:500, Thermo-Fisher) with Alexa Fluor 568-conjugated goat anti-rat IgG (1:500, Thermo-Fisher, Waltham, MA) and anti-rabbit Iba-1 antibody (1:500, Wako) with Alexa Fluor 488-conjugated goat anti-rabbit IgG (1:500, Thermo-Fisher). Sections were then counterstained with DAPI (1:10,000, Thermo-Fisher)^57^. Images were acquired at 20 × magnification, and cells were counted in the perilesional and corresponding, non-injured contralateral regions of two serial sections in the zone with the largest lesion (bregma -1.64 mm and bregma -1.82 mm, www.mbl.org, ROI: 2.55 mm^2^)^55^. In the predefined areas (border zone at lower outside corner the lesion and the corresponding contralateral side) double-immunolabeled cells were counted by an investigator blinded to treatment using ImageJ (U.S. National Institutes of Health, Bethesda, MD).

### RNA isolation and quantitative polymerase chain reaction (qPCR)

For mRNA analysis brain tissue samples were taken from the trimming slices. The right upper quadrant (lesioned brain tissue) of coronal trimming sections were collected (n = 40) as previously described^52^, frozen in liquid nitrogen, and stored at −80 °C^58^. RNA extraction, reverse transcription, and mRNA quantification by real-time RT-PCR were performed as described^53,56^. Absolute copy numbers of target genes were normalized to the reference gene cyclophilin A (*PPIA*).

### Brain water content

Brains were removed 24 h after trauma and placed in a mouse brain matrix. Brains were cut half along the interhemispheric plane and 6 mm slides was taken containing the lesion or the corresponding contralateral side (n = 30), weighed (wet weight, WW), dried in a vacuum-centrifuge (Univapo 100H, UniEquip, Planegg, Germany) for 48 h, and reweighed (dry weight, DW). Brain water content was calculated according to the equation water content (%) = (WW—DW)/WW × 100^48^.

### IgG quantification

The dry-frozen tissue samples (after vacuum-drying to determine the brain water content) were stored at −80 °C. Samples were lysed in RIPA buffer (n = 30), and total cellular proteins separated by SDS-PAGE, transferred to nitrocellulose membranes, immunolabeled (IgG; 1:10 000; Li-cor), and visualized by a near-infrared laser scanning system (LI-COR Odyssey) essentially as described^58^. An antibody against GAPDH (Acris, clone 6C5) was used as the reference and quantification was performed using ImageJ (NIH, MD).

### Statistics

**Figure 4:**
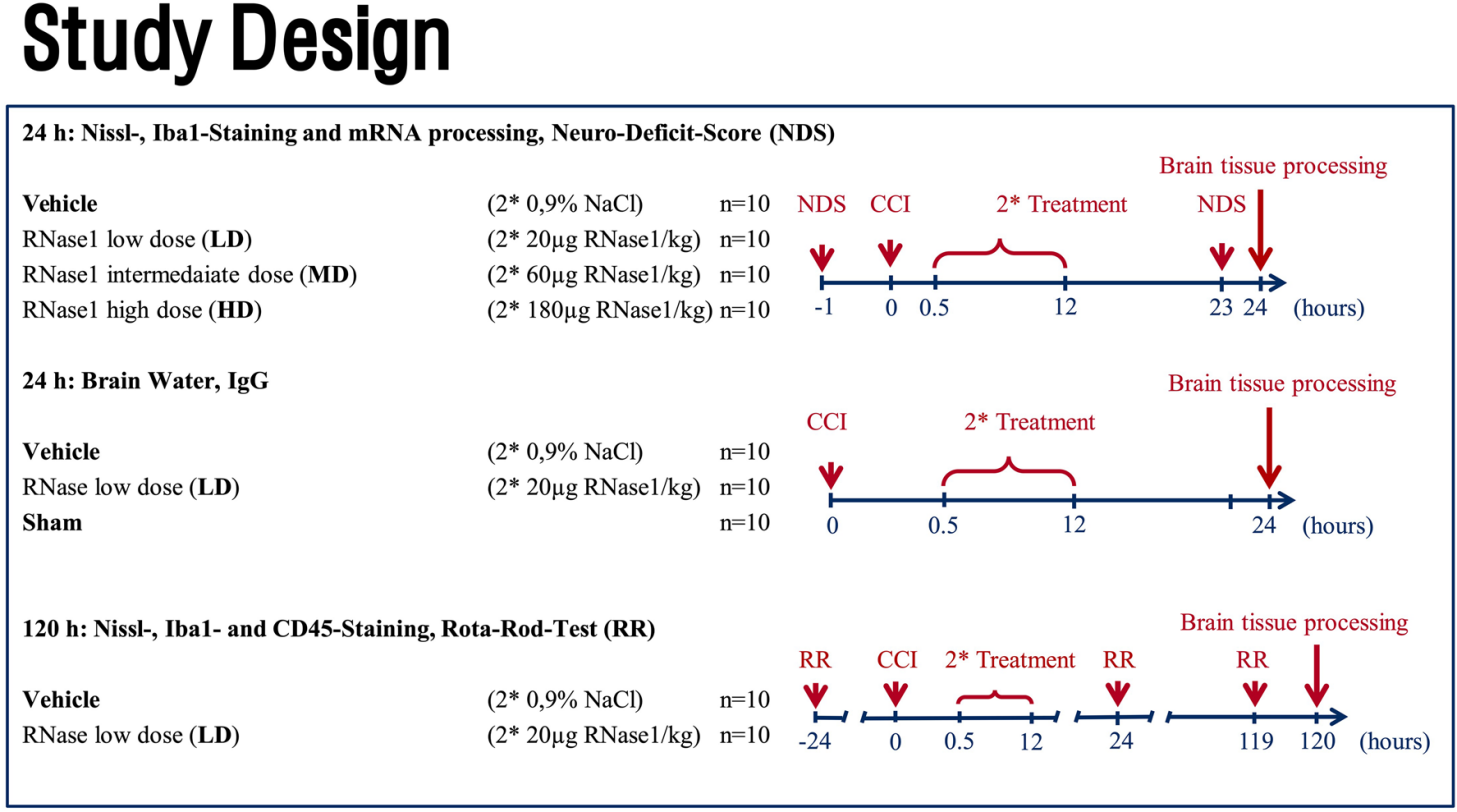
Study Design. The study was performed with 90 male mice. Only the most effective (low) dose of RNase1 was used for subsequent studies of brain water accumulation, tissue IgG changes, and 120-h survival. The figure was generated with Microsoft PowerPoint.

All experiments were randomized and performed by blinded investigators (computer-based randomization). The experimental groups are presented in Fig. 4. To determine the required sample size, an a priori power analysis was performed using G ∗ Power^59^ based on lesion volume data from previous studies. A priori power analysis for an effect size of 0.7 suggested that a standard statistical power (1−β) of Pβ = 0.95 and significance level (α) of 0.05 can be obtained for expected lesion volumes using 10 subjects per group (4 groups) and for expected brain water content using 10 subjects per group (2 groups). GraphPad Prism 9 statistical software (GraphPad Software Inc., La Jolla, CA, USA) was used to perform statistical analysis. Prior to analysis, we checked the test assumptions. Due to the limited power in small samples, we did not perform formal goodness-of-fit tests prior to the t test or ANOVA, but instead relied on the graphical assessment of distribution characteristics^60^. Normality was checked by inspecting the unimodality and symmetry of histograms, as well as by Q-Q plots. The equality of variances was checked by inspecting histograms and standard deviations. For comparison of multiple independent groups, Brown-Forsythe and Welch ANOVA with post-hoc Dunnett T3 multiple comparisons test (comparisons between all groups) was employed. To evaluate group differences in repeated measurements from the same animals (rotarod), RM two-way ANOVA was applied (factors: treatment and time), followed by Šidáks multiple comparisons test. Comparisons between two independent groups were carried out by the Welch’s t test. Values of p < 0.05 were considered significant. Data are presented as the mean and standard deviation (mean ± SD).

## Data sharing

All datasets generated and analyzed during this study are kept in the Dept. of Anesthesiology, Medical Center of the Johannes Gutenberg-University and are available from the corresponding author upon reasonable request.

## Supplementary Information

Supplementary Information 1.
