# Supplementary figures and images for: Ribonuclease-1 treatment after traumatic brain injury preserves blood–brain barrier integrity and delays secondary brain damage in mice
Source: Sci Rep. 2022 Apr 6;12:5731. doi: 10.1038/s41598-022-09326-2 (PMC8986812; doi:10.1038/s41598-022-09326-2)

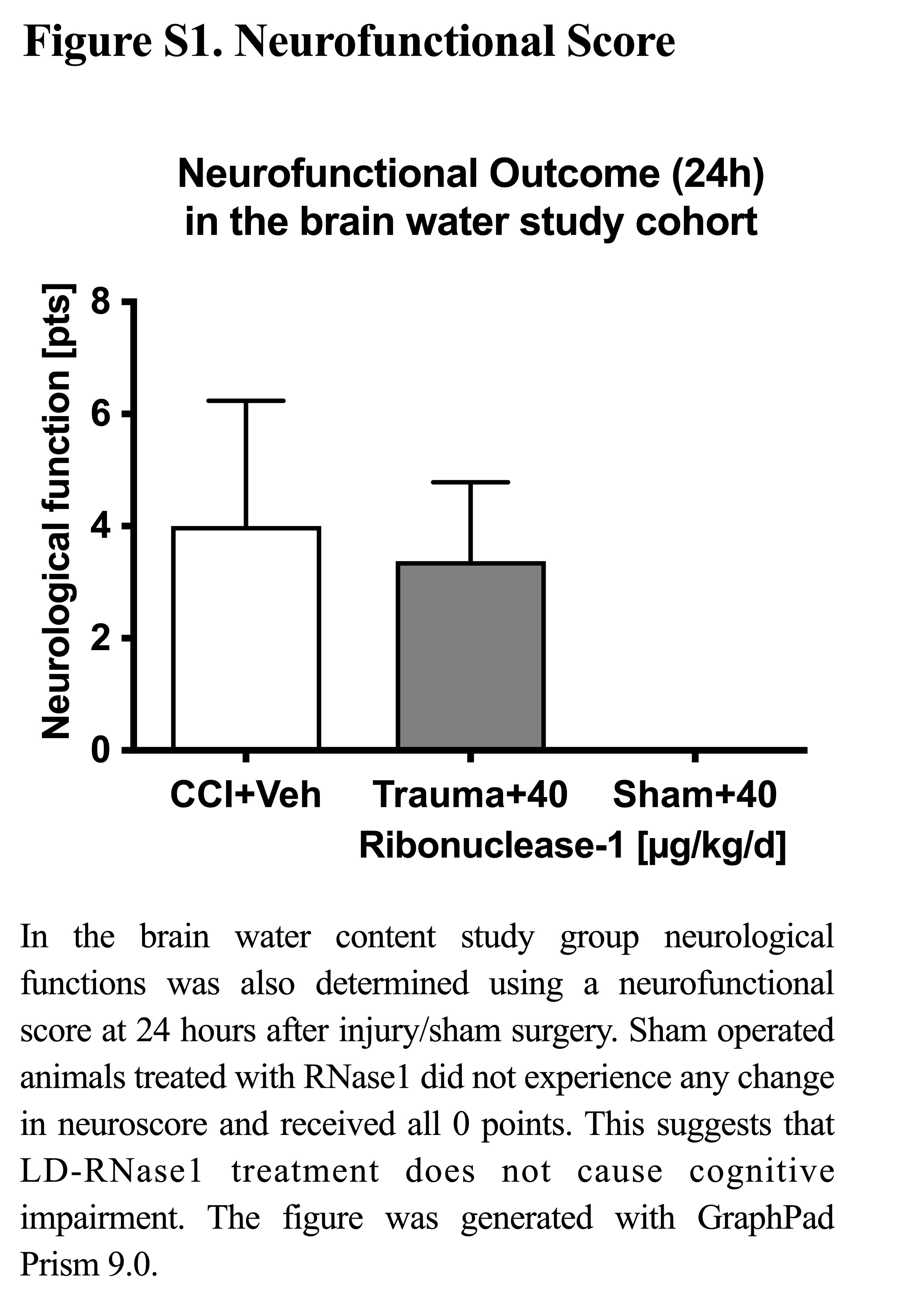

Supplement: Supplementary file 1 — Supplementary Information 1. [file 41598_2022_9326_MOESM1_ESM.jpg]
